# Approaches for peptide and protein cyclisation

**DOI:** 10.1039/d1ob00411e

**Published:** 2021-04-03

**Authors:** Heather C. Hayes, Louis Y. P. Luk, Yu-Hsuan Tsai

**Affiliations:** School of Chemistry, Cardiff University Cardiff CF10 3AT UK; Cardiff Catalysis Institute, School of Chemistry, Cardiff University Main Building Park Place Cardiff CF10 3AT LukLY@cardiff.ac.uk; Institute of Molecular Physiology, Shenzhen Bay Laboratory Shenzhen 518132 China tsai.y-h@outlook.com

## Abstract

The cyclisation of polypeptides can play a crucial role in exerting biological functions, maintaining stability under harsh conditions and conferring proteolytic resistance, as demonstrated both in nature and in the laboratory. To date, various approaches have been reported for polypeptide cyclisation. These approaches range from the direct linkage of N- and C- termini to the connection of amino acid side chains, which can be applied both in reaction vessels and in living systems. In this review, we categorise the cyclisation approaches into chemical methods (*e.g.* direct backbone cyclisation, native chemical ligation, aldehyde-based ligations, bioorthogonal reactions, disulphide formation), enzymatic methods (*e.g.* subtiligase variants, sortases, asparaginyl endopeptidases, transglutaminases, non-ribosomal peptide synthetases) and protein tags (*e.g.* inteins, engineered protein domains for isopeptide bond formation). The features of each approach and the considerations for selecting an appropriate method of cyclisation are discussed.

## Abstract 2

Polypeptide cyclisation can enhance thermal stability, proteolytic resistance and membrane permeability. Cyclisation can be achieved by methods including chemical, enzyme and protein tag approaches. Each has strengths and limitations.

## Graphical abstract

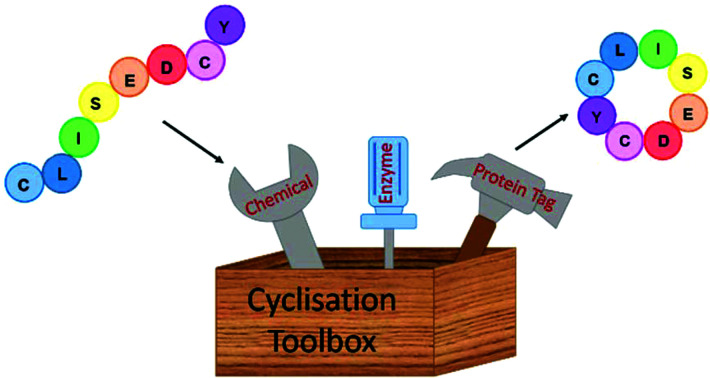

## 1. Introduction

Polypeptides are important biological molecules for all living systems. Most peptides and proteins are linear polymers composed of the 20 canonical amino acids connected through amide bonds. Loosely speaking, peptides are defined as linear chains of ≤50 amino acid residues and proteins of >50 residues.

In the human body and in many other animals, peptides are used as hormones for signal transduction (*e.g.* insulin), whereas proteins are indispensable for cellular structure and function. Indeed, peptides and proteins are closely associated with nearly all human diseases, and thus they have been utilised for disease prevention or treatment.^1–4^ In particular, peptides have become increasingly popular as therapeutics due to their high specificity, high activity and low toxicity.^5^ On a different note, proteins that catalyse reactions have been extensively employed in both research and industrial applications.^6^ These biocatalysts are attractive alternatives to traditional chemical catalysts owing, in particular, to their substrate specificity, catalytic efficiency and biocompatibility. Towards enhancing the biophysical properties of peptides and proteins, as well as expanding their scope of application as therapeutics and catalysts, cyclisation of peptides and proteins has become a burgeoning field of research.

**Fig. 1:**
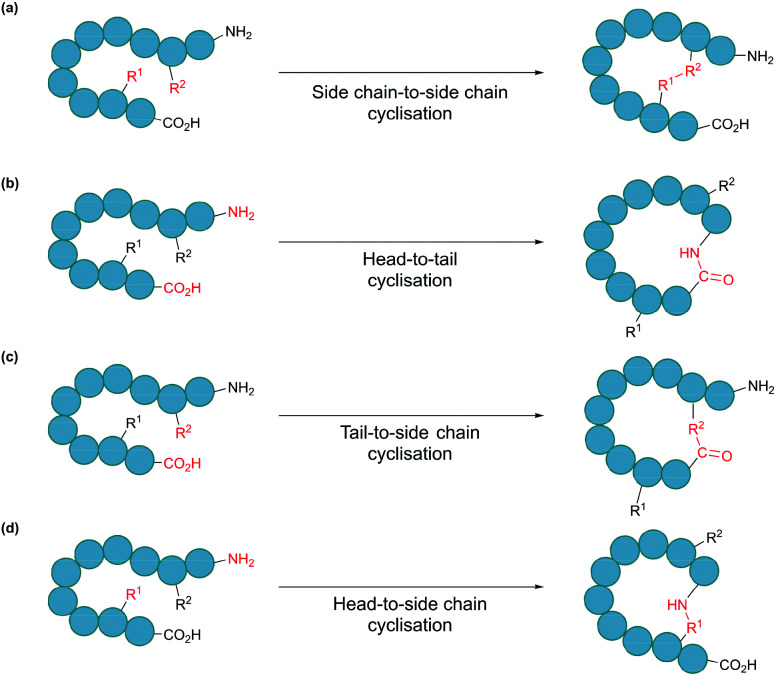
Schematic illustration of cyclisation modes: (a) side chain-to-side chain, (b) head-to-tail, also known as backbone cyclisation, (c) tail-to-side chain, and (d) head-to-side chain.

In nature, many polypeptides are found to be cyclised, a feature that is often intrinsically associated with their biological function. Cyclisation can be categorised into four general classes: side chain-to-side chain, head-to-tail (also known as backbone cyclisation), head-to-side chain and side chain-to-tail (Fig. 1). Side chain-to-side chain cyclisation occurs when a bond is formed between the side chain functionalities of two amino acid residues (Fig. 1a). One prominent example is intramolecular disulphide bond formation between the thiol functionalities of two cysteine residues, leading to a type of cyclic structure, commonly found in peptides and proteins such as insulin and antibodies. It is estimated that about 50% of cysteine residues in polypeptides are found in the form of disulphide bonds.^7^ Other types of side chain cyclisation, including non-native linkages, are also possible and will be discussed throughout this review. Head-to-tail terminus cyclisation is another commonly observed form of cyclisation. As the first residue in a chain of amino acids has an amino functionality (*i.e.* N-terminus), and the last residue has a carboxylate functionality (*i.e.* C-terminus), polypeptides are typically directional. Subsequently, cyclisation can be achieved by joining the N- and C-termini through an amide bond (Fig. 1b). Head-to-tail peptide cyclisation has been observed in microorganisms and plants, such as kalata B1 from the plant *Oldenlandia affinis* and bacteriocin AS-48 produced by the bacterium *Enterococcus faecalis*.^8,9^ Furthermore, a recent report shows that head-to-tail cyclic peptides are prevalent in normal flora such as those in the human gut.^10^ Meanwhile, the formation of a lactam, lactone or thiolactone between either terminus with an appropriate side chain functional group (Fig. 1c and d) results in side chain-to-terminus cyclisation. For example, bacitracin is an antibiotic side chain-to-tail cyclic peptide produced by *Bacillus subtilis*, in which a bond is formed between a lysine side chain and the C-terminus.^11^

As potential drugs, both natural and synthetic peptides are increasingly researched due to their favourable characteristics.^12^ However, they often suffer from low oral bioavailability and metabolic instability. These shortcomings can often be addressed by cyclisation. Firstly, peptide cyclisation can lead to improved biological activity by enabling enhanced binding towards the target molecule. As a result of decreased conformational flexibility, the more rigid macrocycle has a reduced change in entropy upon binding to the target molecule compared to that of the linear peptide.^13,14^ Secondly, head-to-tail cyclic peptides have increased resistance to hydrolysis by exopeptidases due to the absence of the free termini. Thirdly, membrane permeability and cytoplasmic delivery of the molecule is enhanced, though the exact mechanism remains not entirely clear.^15^ Consequently, the favourable pharmacological properties possessed by cyclic peptides makes them attractive therapeutic candidates. Indeed, nine cyclic peptide drugs were approved for market between 2006 and 2015.^12^ While the majority of cyclic peptides in clinical use are currently derived from natural sources, the design of synthetic cyclic peptide drug candidates is becoming increasingly common, aided by advances in computational design and high-throughput screening.^16^

For proteins, cyclisation can be employed to improve stability so that enzymes can function outside of their native conditions, such as at elevated temperatures, in acidic or basic environments and in the presence of organic solvents or additives.^15,17,18^ According to polymer theory, the overall increase in the stability of a cyclised protein originates from the destabilisation of the unfolded state.^19^ Upon cyclisation, the number of conformational states accessible to the unfolded polypeptide is reduced. As a result, the entropy of the unfolded state is decreased and, consequently, the Gibbs free energy is increased. It should be noted that an offsetting enthalpic cost may occur if cyclisation introduces strain to the system, in which case, the desired stabilisation may not be achieved.^20^ Although the stabilisation of industrially relevant enzymes usually relies on techniques such as directed evolution and computational design,^21^ a number of proteins have been shown to benefit from cyclisation. These include β-lactamase, dihydrofolate reductase and luciferase, all of which exhibit improved activity at elevated temperatures compared to their linear forms.^22–24^

In this review, we first introduce different approaches for peptide and protein cyclisation. Broadly speaking, these approaches can be categorised as either chemical, enzymatic or protein tag methods. While each approach has its own strengths and limitations, the choice of the most appropriate approach largely depends on factors such as the desired application and means to produce the material. Additional considerations will be discussed in the latter part of the review.

## 2. Chemical methods for cyclisation

Many chemical ligation methods have been developed over the years, which when applied in an intramolecular manner to the peptide or protein of interest, result in cyclisation. The reader should note that in this review the term ligation refers to bond formation between two polypeptide functional groups, whether this is in an inter- or intramolecular manner. Meanwhile, the term cyclisation specifically refers to intramolecular ligation resulting in one of the four classes of cyclic polypeptide products discussed in section 1 (Fig. 1). In this section, we briefly discuss the strengths and limitations of some important traditional chemical cyclisation methods. We then highlight recent examples that have built on these methods and address some of their shortcomings. It should be noted that examples discussed here are in no way exhaustive, and we direct readers interested in this area to more comprehensive reviews of this subject.^25–28^

### 2.1. Direct amide bond formation

Amide bond formation between carboxylic acid and amine groups can take place *via* direct condensation using high temperatures or microwave irradiation.^29,30^ However, such harsh conditions are incompatible with most peptides and proteins. Thus, reactions with milder conditions have been developed for polypeptide ligation. This is commonly achieved by transforming the C-terminal –OH into a better leaving group, such as an acyl halide, acyl azide, anhydride or an activated ester, through the use of coupling reagents.^31^ Accordingly, nucleophilic (*e.g.* Lys, Ser, Thr) and carboxylate (*e.g.* Asp, Glu) amino acid side chains must be protected to prevent side reactions, and thus this approach is more suitable for peptides that are synthesised in a fully protected form. In addition, additives are often used in combination with coupling reagents to suppress racemisation at the ligation site and enhance the rate of reaction.^32^ For example, in the total synthesis of a 13 residue depsicyclic peptide antibiotic, texiobactin, the cyclisation step was successfully carried out using a combination of coupling reagents (HOAt/OxymaPure/HATU) with a tertiary amine base DIEA.^33^ However, alternative cyclisation methods that do not require protecting strategies and that can be carried out under physiological conditions are often preferred.

### 2.2. Native chemical ligation

**Fig. 2:**
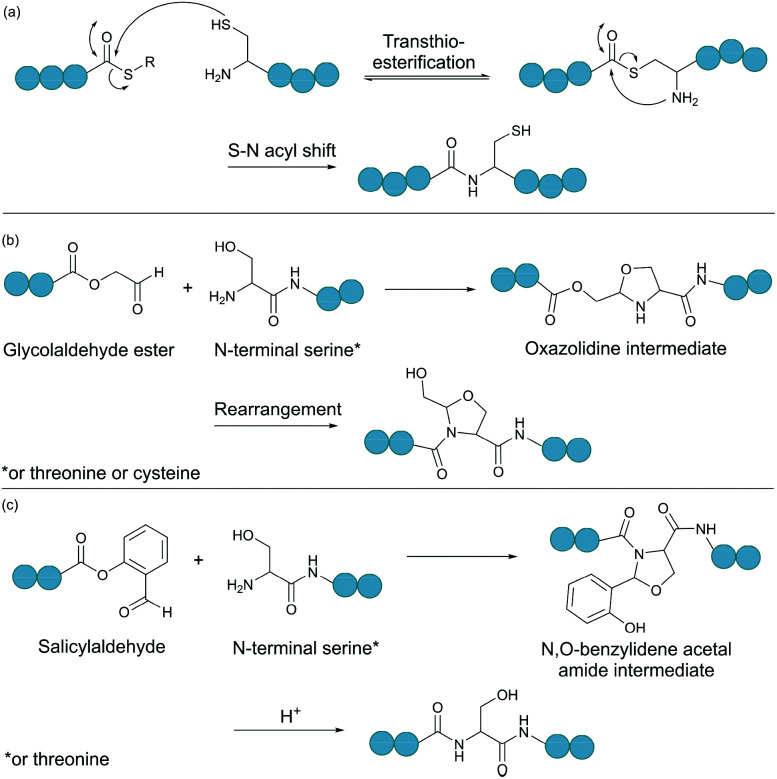
Selected chemical ligation methods. (a) Peptide bond formation by native chemical ligation. (b) Bond formation between a N-terminal Ser/Thr/Cys and a C-terminal glycolaldehyde. (c) Ser/Thr ligation with a C-terminal salicylaldehyde.

Native chemical ligation (NCL) is as an effective method for linking two unprotected peptide fragments (Fig. 2a).^34,35^ One fragment contains a C-terminal thioester, and the other contains an N-terminal cysteine residue. Both fragments can be produced either chemically by solid-phase peptide synthesis or recombinantly from cells (see section 4.1 for the recombinant introduction of C-terminal thioesters). Importantly, the reaction proceeds in aqueous conditions at neutral pH, and tolerates the presence of chaotropic reagents (*e.g.* guanidine hydrochloride) as well as reducing agents. Mechanistically, the reaction begins with the nucleophilic attack of the thiol group of the N-terminal cysteine to the carbonyl carbon of the thioester group, leading to rapid and reversible transthioesterification. Subsequently, an S-to-N acyl shift generates the desired peptide bond. This reaction is regio- and chemoselective, as neither the presence of internal cysteine residues nor other nucleophilic amino acid side chains interfere with the reaction.

Backbone cyclisation can be achieved by intramolecular NCL. This was first demonstrated by Zhang and Tam with peptides ranging from 5 to 16 residues. They observed a high yield of cyclic product with no oligomer formation from intermolecular reaction, even at millimolar concentrations.^36^ More recently, a series of cyclic peptides between 10 and 28 amino acid residues, were prepared using a microfluidic NCL procedure.^37^ This enabled acceleration of the NCL reaction, with fast ligation observed even with less reactive C-terminal thioesters, such as those on Val, Ile or Pro. The microfluidic NCL strategy was also successfully employed for the preparation of an 18-residue cyclic peptide RTD-1, which displayed antibacterial activity against *E. coli* and *S. aureus*.

Over the years, a variety of other extensions to the NCL methodology have been devised, expanding the scope of application.^35^ For example, post-ligation desulphurisation using a free-radical and metal-free reduction method can convert cysteine into alanine.^38^ The removal of the cysteine and replacement by a much more abundant amino acid is advantageous, as the desired polypeptide of interest can be generated with no trace of ligation or mutation. The development of other thiol-containing unnatural amino acids has further increased the number of sites possible for the ligation reaction to take place.^39^ Another important extension to the NCL method includes the use of selenium in place of sulphur to accelerate the rate of the ligation reaction and minimise side reactions such as thioester hydrolysis.^34,35,40^

### 2.3. Ligations relying on a C-terminal aldehyde

Serine and threonine residues can also be employed for ligating unprotected peptide fragments, and hence cyclisation. For example, a C-terminal glycolaldehyde ester reacts chemoselectively with an N-terminal serine or threonine (or cysteine) residue to form an oxazolidine intermediate, which after rearrangement generates a peptide bond in the form of pseudoproline structure (Fig. 2b).^41^ However, the reaction is slow and leaves an unnatural functionality at the ligation site. To overcome these limitations, a modified approach using a C-terminal salicylaldehyde was developed.^42^ After oxazolidine formation from the chemoselective reaction of the salicylaldehyde with the N-terminal Ser/Thr, an *N*,*O*-benzylidene acetal amide intermediate is generated upon O–N acyl shift. Using TFA the acyl group can then be removed, leaving a native peptide bond at the ligation site (Fig. 2c). This Ser/Thr ligation approach has been successfully applied to the synthesis of a number of cyclic peptide natural products, including daptomysin,^43^ cyclomontanin B,^44^ mahafacyclin B,^45^ among others.^46,47^

**Fig. 3:**
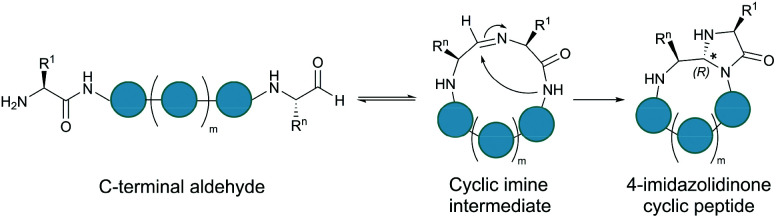
Peptide cyclisation by CyClick involving the N-terminal amine and the C-terminal aldehyde (5 ≤ *n* ≤ 9, *m* = *n* − 4).

A C-terminal aldehyde functionality is also employed in a recently developed cyclisation, known as CyClick (Fig. 3).^48^ Cyclisation proceeds with the reaction of the C-terminal aldehyde and the N-terminal amine, forming a cyclic peptide with an imine intermediate. The imine group is subsequently attacked by the adjacent amide nitrogen atom to form an imidazolidinone. The final bicyclic product is thermodynamically stable, driving the reaction toward intramolecular cyclisation. This reaction could be performed at concentrations up to 100 mM without significant production of dimer or oligomer side products from intermolecular reactions. It is noteworthy that the ε-amine of lysine does not interfere with CyClick. Though being efficient, highly chemoselective and stereoselective, CyClick has only been applied to synthetic peptides due to the need for a C-terminal aldehyde functionality.

### 2.4. Bioorthogonal reactions

A bioorthogonal reaction involves two complementary bioorthogonal functionalities, which do not react with naturally occurring biological molecules, but selectively with each other under physiological conditions.^49^ Thus, the use of bioorthogonal reactions for peptide and protein modification eliminates concerns about side reactions and off-target effects.

**Fig. 4:**
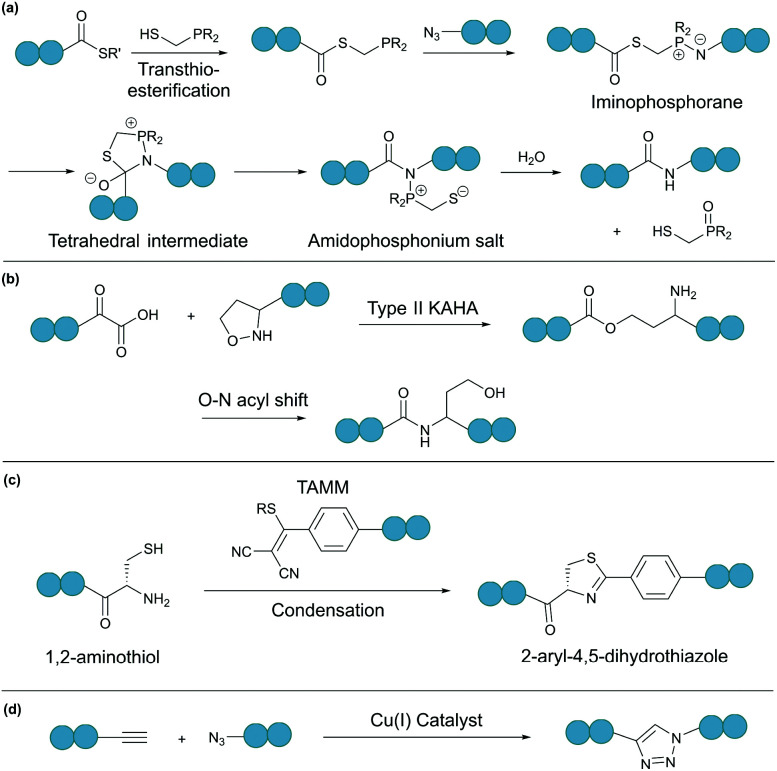
Selected bioorthogonal reactions for peptide/protein cyclisation: (a) traceless Staudinger ligation, (b) type II KAHA ligation, (c) TAMM condensation, and (d) CuAAC.

Theoretically, any bioorthogonal reaction can be used for peptide and protein cyclisation, although site-specific introduction of the required bioorthogonal functionalities is the prerequisite. For instance, Staudinger ligation is based on the reduction of an azide into an amine by a phosphine. For traceless backbone cyclisation, a bifunctional phosphinothiol reagent was developed (Fig. 4a).^50^ The ligation begins with a transthioesterification reaction between the peptide thioester and phosphinothiol, followed by reaction with the other peptide fragment bearing an N-terminal azide. The resulting iminophosphorane intermediate is then transformed into an amidophosphonium salt through a cyclic tetrahedral intermediate. Hydrolysis of the amidophosphonium salt produces a native amide bond between the peptide fragments. While the reaction has been successfully applied for cyclising synthetic peptides,^51^ the application of the Staudinger ligation in aqueous solutions is limited due to the laborious preparation of water-soluble phosphinothiols.^52^ The widespread application of the Staudinger ligation has further been hindered by the requirement for substrates with glycine residues at the ligation site. Due to increased steric strain on the tetrahedral intermediate in the presence of bulkier residues, a covalent bond between the oxygen and the oxophilic phosphorous atom is favoured over thiol displacement. However, by increasing the electron density on the phosphorous atom of the phosphinothiol reagent, P–O bond formation is discouraged and an improved yield can be achieved for non-glycyl Staudinger ligation reactions.^53^

In a second example, an amide bond can be formed by α-ketoacid-hydroxylamine (KAHA) ligation.^54^ This chemoselective ligation takes place between an N-terminal hydroxylamine and a C-terminal α-ketoacid. Depending on the nature of the hydroxylamine substituent, there are two main mechanisms. Type I KAHA ligation uses a free hydroxylamine, while type II KAHA involves the use of an *O*-substituted hydroxylamine. Though demonstrated to be a feasible method for the cyclisation of medium length peptides, type I KAHA is rarely used, mainly due to the instability of the free hydroxylamine in aqueous media.^55^ For the type II KAHA ligation, a water stable *O*-substituted hydroxylamine, most commonly 5-oxaproline, can be easily prepared and incorporated using solid-phase peptide synthesis. After cleavage from the resin, the α-ketoacid and 5-oxaproline cyclise directly, generating a depsipeptide intermediate, which under basic conditions, undergoes O–N acyl shift to give the desired cyclic product, ligated by an amide bond (Fig. 4b).^56^ A homoserine residue is formed at the ligation site, however, by replacement of the N-terminal oxaproline with an oxazetidine functionality, a serine residue is instead produced upon KAHA ligation.^57^ Type II KAHA ligation has been used to cyclise a variety of short peptides.^58^ Furthermore, this approach was shown to be compatible with larger and more challenging substrates, as demonstrated by the chemical synthesis of the cyclic antibacterial protein AS-28, which has 70 amino acid residues.^59^

Recently, we reported a novel bioorthogonal reaction involving 1,2-aminothiol and 2-((alkylthio)(aryl)methylene)malononitrile (TAMM) (Fig. 4c).^60^ The TAMM functionality is stable over a range of pH values and temperatures. Though an unnatural moiety remains in the product, this reaction is fast (*k* ∼10 M^−1^ s^−1^) and specific with no cross reaction with internal cysteine or other nucleophilic residues observed. Using this reaction, cyclisation of proteins on bacteriophages was demonstrated with no reduction in phage infectivity observed.

The bioorthogonal reactions mentioned thus far all require non-native functionalities, which can be readily introduced using solid-phase peptide synthesis or in some instances by recombinant approaches.^49,61^ For example, the technique of genetic code expansion enables site-specific incorporation of unnatural (non-canonical) amino acids that contain a bioorthogonal functionality.^62,63^ To do this, a blank codon (usually the amber stop codon, UAG) and an orthogonal aminoacyl-tRNA synthetase/tRNA pair are required. The orthogonal synthetase does not recognise any endogenous tRNA or canonical amino acids as its substrate, and the orthogonal tRNA is not a substrate of any endogenous synthetases. The orthogonal synthetase specifically loads the orthogonal tRNA with the designated unnatural amino acid. This aminoacylated tRNA recognises the blank codon on the mRNA and directs the site-specific incorporation of the unnatural amino acid into the target protein.^62,63^

Chin and co-workers have demonstrated the use of copper-catalysed azide–alkyne cycloaddition (CuAAC) (Fig. 4d) for protein cyclisation through genetically incorporated azide- and alkyne-containing amino acids.^64^ By definition, “click”-type reactions, such as CuAAC, are simple, rapid, high yielding, stereospecific and wide in scope.^65^ As such, CuAAC has been used extensively for peptide cyclisation,^66^ which can be carried out under mild conditions in a variety of solvents, including water.^67,68^ The 1,4-disubstituted triazole formed at the ligation site, although not a natural functionality of polypeptides, is known to effectively mimic the topology and electronic properties of native *trans*-amide bonds.^69^ Theoretically, CuAAC can be employed for peptide and protein cyclisation in live cells through the use of appropriate ligands that can increase the reaction rate and reduce catalyst toxicity.^70,71^

In place of a terminal alkyne, a strained alkyne can react with an azide in the absence of a catalyst under physiological conditions.^72^ However, this strain promoted azide–alkyne cycloaddition (SPAAC) lacks the regiospecificity of CuAAC, and forms a mixture of 1,4-disubstituted products. Furthermore, the synthesis of strained alkynes is more laborious.^73^ Nevertheless, a peptide cyclised using SPAAC exhibited improved proteolytic stability and binding affinity compared to its linear peptide counterpart.^74^

Alternatively, through the use of a ruthenium(ii) catalyst, the 1,5-disubstituted regioisomer is generated upon azide–alkyne cycloaddition (RuAAC).^75,76^ The 1,5-disubstituted triazole mimics a *cis*-amide bond and is beneficial to cyclisation of peptides not only as the cyclisation machinery, but also when introduced into the peptide chain before cyclisation, it acts as a turn inducer to bring the linear termini into close proximity for ligation.^77^ In addition, ruthenium catalysts can also be used for ring-closing metathesis, which was successfully utilised for the stabilisation of α-helices through peptide stapling.^78,79^ For more detailed discussions on metal-catalysed polypeptide cyclisation we direct the interested readers to references.^80–82^

### 2.5. Disulphide bonds

**Fig. 5:**
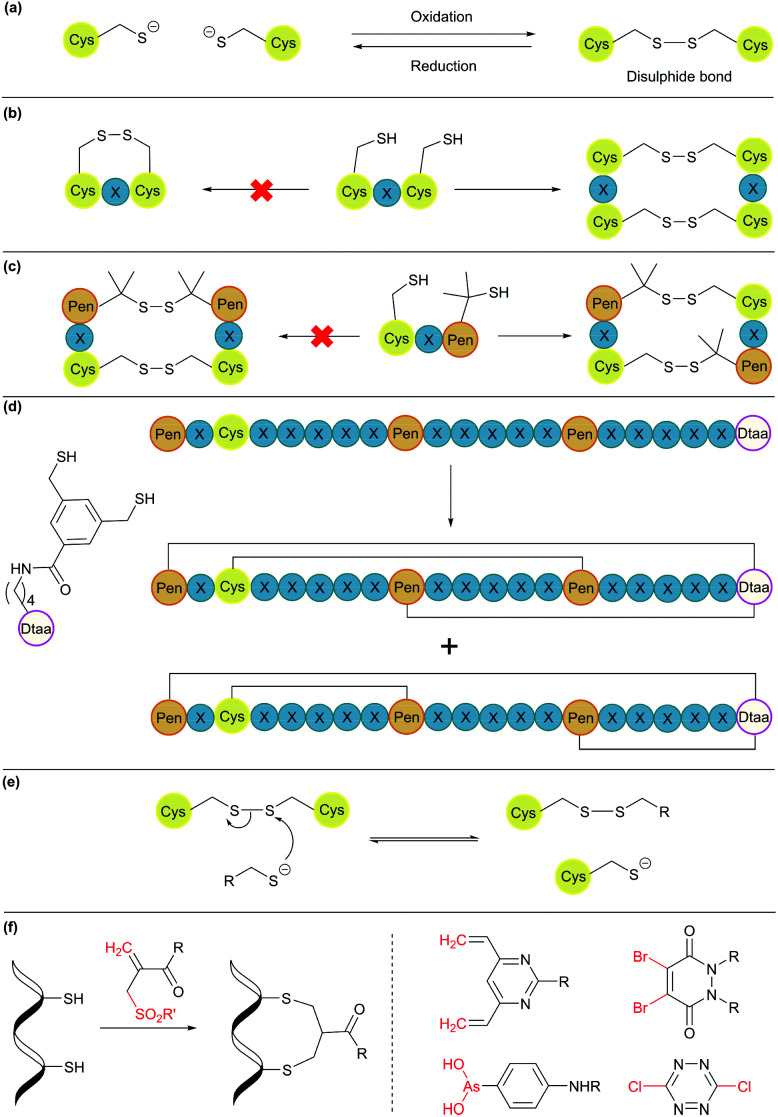
Cyclisation through disulphide bonds. (a) A disulphide bond is formed and cleaved under oxidative and reductive conditions, respectively. (b) Orthogonal disulphide pairing using the CXC motif (X = any amino acid), which does not form a disulphide bond between two Cys residues separated by one amino acid. (c) In the presence of penicillamine (Pen) residues, Pen and Cys form thermodynamically more stable mixed disulphide bonds. (d) Use of an unnatural dithiol amino acid, Dtaa, to moderate the number of possible disulphide patterns. (e) A thiol-containing molecule can react with a disulphide molecule *via* the thiol-disulphide exchange reaction. (f) Disulphide stapling reagents that react with two Cys residues and form a stable covalent adduct. Selected stapling reagents are shown on the right.

Disulphide bond formation is arguably the most common chemical method for cyclisation (Fig. 5a). About 30% of eukaryotic proteins have at least one disulphide bond, which can stabilise the protein three-dimensional structure and regulate protein function.^7^ Cysteine residues can be easily introduced into amino acid chains by either chemical or recombinant means, and disulphide bonds usually form spontaneously upon exposure to air. This concept has been used to engineer proteins with a cyclic structure by introducing two cysteine residues.^18^ However, for proteins with several cysteine residues, there are many possible disulphide bond patterns. For example, a protein with 6 cysteine residues can form 3 intramolecular disulphide bonds in 15 (5 × 3 × 1) different ways. While cells produce enzymes to ensure the formation of the correct disulphide bond pattern,^83^ the process can be difficult to control in reaction vessels and often leads to a mixture of products.

Orthogonal cysteine protecting groups can be employed to facilitate regioselective disulphide bond formation in cysteine-rich peptides.^84^ Alternatively, to overcome the high number of possible disulphide bond patterns, orthogonal disulphide pairing has been developed. This concept relies on a unique sequence of cysteine residues or unnatural side chain functionalities to reduce the number of possible disulphide bond patterns. For example, the two cysteine residues in a CXC motif (X = any amino acid) do not form a disulphide bond with each other. Instead, formation of two disulphide bonds between two CXC motifs is preferred (Fig. 5b).^85^ Moreover, if penicillamine (Pen) is present, formation of a mixed disulphide bond is thermodynamically preferred over disulphide bond formation between two cysteine residues or two penicillamine residues (Fig. 5c).^86^ However, no disulphide bond is formed in Pen–X–C motifs, just like CXC motifs. By combining these two orthogonal disulphide pairing strategies, it was demonstrated that for a series of peptide sequences containing six thiol functional groups, the number of isomers obtained was as low as four, and in some cases, only one specific isomer was isolated.^87^ Similarly, a selenocysteine residue or an unnatural dithiol amino acid (Dtaa) can also be used to moderate the number of possible disulphide patterns (Fig. 5d).^88,89^

While orthogonal disulphide pairing is effective in minimising the number of isomers generated, their use is generally limited to synthetic materials due to the requirement of unnatural amino acids such as penicillamine. Furthermore, disulphide bonds are not stable under reductive environments (*e.g.* cytosol) and can be disrupted by thiol-containing molecules through a thiol-disulphide exchange reaction (Fig. 5e).^90^

Disulphide stapling reagents have addressed the stability issue. A stapling reagent normally contains two electrophiles, such as arsenous acid derivatives,^91^ dibromopyridazinediones,^92^ disubstituted maleimides,^93,94^ perfluoroaryl deriviatives,^95^ among others (Fig. 5f).^96,97^ These molecules can be subjected to two nucleophilic additions or substitutions by two thiol groups. Thus, after a disulphide bond is reduced, the addition of a disulphide stapling reagent re-bridges the side chain of two cysteine residues as more stable thioether linkages.

### 2.6. Remarks

In summary, the most common limitation for chemical cyclisation methods is the need for functionalities not provided by the 20 canonical amino acids. Nevertheless, some approaches, such as cyclisation by disulphide bond formation, do employ canonical amino acids. In addition, advances in genetic code expansion have enabled the site-specific incorporation of some unnatural amino acids with the required functionalities. This has allowed the recombinantly produced materials to be subjected to chemical cyclisation approaches, as well as the application of chemical approaches in living systems.

## 3. Enzymatic methods for cyclisation

Enzymes are known for their efficiency and selectivity in catalysing reactions under mild conditions. In addition, an enzyme can often catalyse both the forward and the reverse reactions. For example, proteases are enzymes that catalyse amide bond hydrolysis, but they can also catalyse amide bond formation. Indeed, there has been a long history of using proteases to catalyse peptide bond formation.^98^ However, the reversible nature of enzyme catalysis can prevent the reaction from reaching completion. To increase the yield of the desired ligation product, the reaction equilibrium must be shifted to favour peptide bond formation.^99^ This can be achieved by different tactics, such as the use of thermodynamically less stable starting materials, alteration of the reaction pH, inclusion of co-solvents, or addition of additives. All approaches are theoretically applicable to peptide and protein substrates prepared either chemically or recombinantly.

### 3.1. Subtiligase variants

**Fig. 6:**
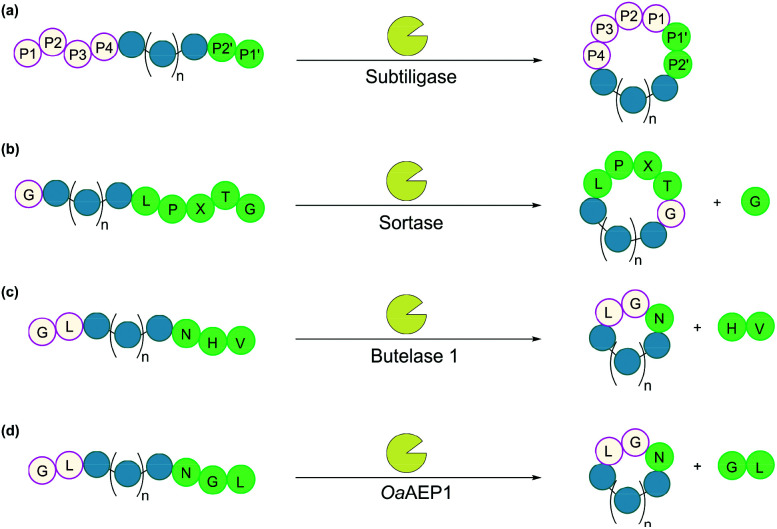
Enzymatic methods for backbone cyclisation (in some cases recognition sequences may vary): (a) subtiligase, (b) sortase, (c) butelase 1, and (d) *Oa*AEP1.

Subtiligase is a double mutant of the serine protease subtilisin BPN’ from *Bacillus amyloliquefaciens*.^100^ It recognises a range of substrate sequences. Moreover, protein engineering has afforded variants that have an even wider substrate scope^101^ or can function independently of Ca^2+^.^102^ The combined efficiency and broad substrate scope make subtiligase variants attractive tools for traceless ligation, forming backbone cyclised peptidyl products (Fig. 6a).^100–102^ Despite these appealing features, C-terminal (thio)ester substrates are required for use by subtiligase variants. Although proteins with a C-terminal thioester can be produced recombinantly using an intein-mediated strategy,^103^ the process is sequence-dependent and often requires lengthy operational steps that result in low yields. Consequently, ester and thioester substrates are often made through chemical synthesis, which limits potential applications.^104^

### 3.2. Sortases

Sortases are cysteine transpeptidases found in many bacterial species, particularly Gram-positive ones. Among those reported, sortase A from *Staphylococcus aureus* (SrtA) is the most widely used for peptide and protein modifications.^105^ SrtA recognises the amino acid sequence LPXTG, where X is any amino acid, cleaving the amide bond between Thr and Gly residues and forming a thioester intermediate at the C-terminus of Thr, before nucleophilic attack by a peptide with a N-terminal Gly (Fig. 6b).

With regard to peptide cyclisation, both the length and concentration of the peptide have been demonstrated to affect the preference of SrtA for backbone cyclisation *versus* oligomerisation.^106^ It was reported that substrates of a minimum length of 19 residues (including the LPXTG motif) are required for cyclisation to be favoured over intermolecular reactions (*i.e.* formation of dimers and trimers in linear or cyclic forms). Increase of peptide concentration (>1 mM) was unsurprisingly accompanied with an increase of intermolecular di- and trimerisation. SrtA has also been used to cyclise larger recombinant proteins, including various cytokines, green fluorescence protein (GFP) and ubiquitin C-terminal hydrolase L3.^107,108^

While SrtA and its variants are valuable tools for peptide and protein modifications, achieving high ligation yields often requires the use of excess amounts of nucleophilic peptide due to the reversible nature of the enzyme. A variety of methods have been developed to circumvent this problem by the removal of small glycyl leaving groups using dialysis,^109^ use of unnatural starting material to enable quenching of the glycyl leaving groups,^110^ or a flow-based system.^111^ Furthermore, recent advances in protein engineering have yielded SrtA variants with >100 fold increase in activity,^112,113^ as well as Ca^2+^-independent variants for cellular applications.^114^

### 3.3. Asparaginyl endopeptidases

Asparaginyl endopeptidases are cysteine proteases that catalyse peptide bond cleavage after an Asx (*i.e.* Asn or Asp) residue. These enzymes are mostly found in plants, many of which are capable of mediating transpeptidation, producing naturally occurring cyclic peptides. Butelase 1 from *Clitoria ternatea* and asparaginyl endopeptidase 1 from *Oldenlandia affinis* (*Oa*AEP1) are the two most prominent examples which have been utilised for peptide and protein cyclisation.^115^

Butelase 1 shows a strong preference for the catalysis of transpeptidation over the hydrolysis of Asx-containing substrates.^116^ Its efficiency enables low enzyme loading (<0.01 eq.), and its broad sequence promiscuity facilitates transpeptidation in a nearly traceless manner. In the butelase 1 reactions, the NHV preferred recognition sequence, is added to the C-terminal of the substrate (Fig. 6c). Meanwhile, for the nucleophilic peptide, the N-terminal sequence should start with either GX or XL (where X is any amino acid, including d-amino acids).^117^ Butelase 1 has been employed for backbone cyclisation of different peptides including the cyclotide kalata B1, sunflower trypsin inhibitor (SFTI), conotoxin MrIA, insect antimicrobial peptide thanatin, antimicrobial peptide histatin-3 and θ-defensin,^116,117^ as well as proteins including GFP, interleukin-1 receptor antagonist and somatropin.^118^

Similar to butelase 1, *Oa*AEP1 exhibits broad sequence promiscuity. In particular, the variant *Oa*AEP1-C247A, in which the peptide-binding domain is modified, was reported to be kinetically superior to that of the wild-type enzyme.^119^ While NGL is its native recognition sequence (Fig. 6d), the Gly residue can be replaced with most other amino acids (except Pro), and the Leu residue can be replaced with other bulky amino acids (*e.g.* Phe, Ile, Met, Trp).^104^ Moreover, *Oa*AEP1 can be easily produced in *E. coli* without lengthy activation or purification steps, unlike many other asparaginyl endopeptidases.^104^ A variety of molecules have been cyclised by *Oa*AEP1,^104,119–121^ including an intrinsically disordered protein, MSP2,^121^ which butelase 1 failed to cyclise.^118^

In addition to butelase 1 and *Oa*AEP1, new asparaginyl endopeptidases are continuously being discovered and employed for peptide and protein cyclisation, some of these include *Ha*AEP1,^122^*MCo*AEP2^123^ and *Vy*PAL1-3.^124^ These advances not only provide more ligation tools but also enhance our understanding of the molecular mechanism of the enzymes, laying the foundation for engineering variants with versatile functions. Theoretically, asparaginyl endopeptidases can be employed in living systems, such as for the modification of cell surface proteins, although this has only been demonstrated with butelase 1.^125^

### 3.4. Transglutaminase

The enzymes discussed so far catalyse head-to-tail cyclisation by amide bond formation between the terminal carboxylic acid and amino groups. There are also enzymes that facilitate bond formation between amino acid side chains. Transglutaminases are a family of enzymes, found in microorganisms, plants and animals, that catalyse an acyl transfer reaction between the carboxyamide group of glutamine residues and various primary amines (including the ε-amino group of lysine residues), with NH_3_ released as a by-product.^126^ The resulting crosslinking amide bond, known as an isopeptide bond, is chemically and proteolytically stable.^127^ A calcium-dependent microbial transglutaminase from *Streptomyces mobaraensis* was used to cyclise a variety of peptide sequences ranging from 11 to 23 amino acids.^128,129^ Broad substrate specificity was observed, although an Ala and Leu dipeptide sequence was required at the N-terminal side of the glutamine residue.^128^ Generally, isopeptide bond formation will occur so long as both substrate lysine and glutamine residues are accessible to the enzyme. However, the sequence of amino acids flanking the glutamine residue may also affect the reaction yield.^130^

Despite having been demonstrated as a useful peptide cyclisation tool, with benefits including its tolerance to a range of temperatures and pH values, irreversible ligation, and commercial availability, there are few examples in the literature of microbial transglutaminase-catalysed cyclisation due to low sequence specificity when the polypeptide of interest contains multiple lysine and glutamine residues.^128^

### 3.5. Non-ribosomal peptide synthetases

**Fig. 7:**
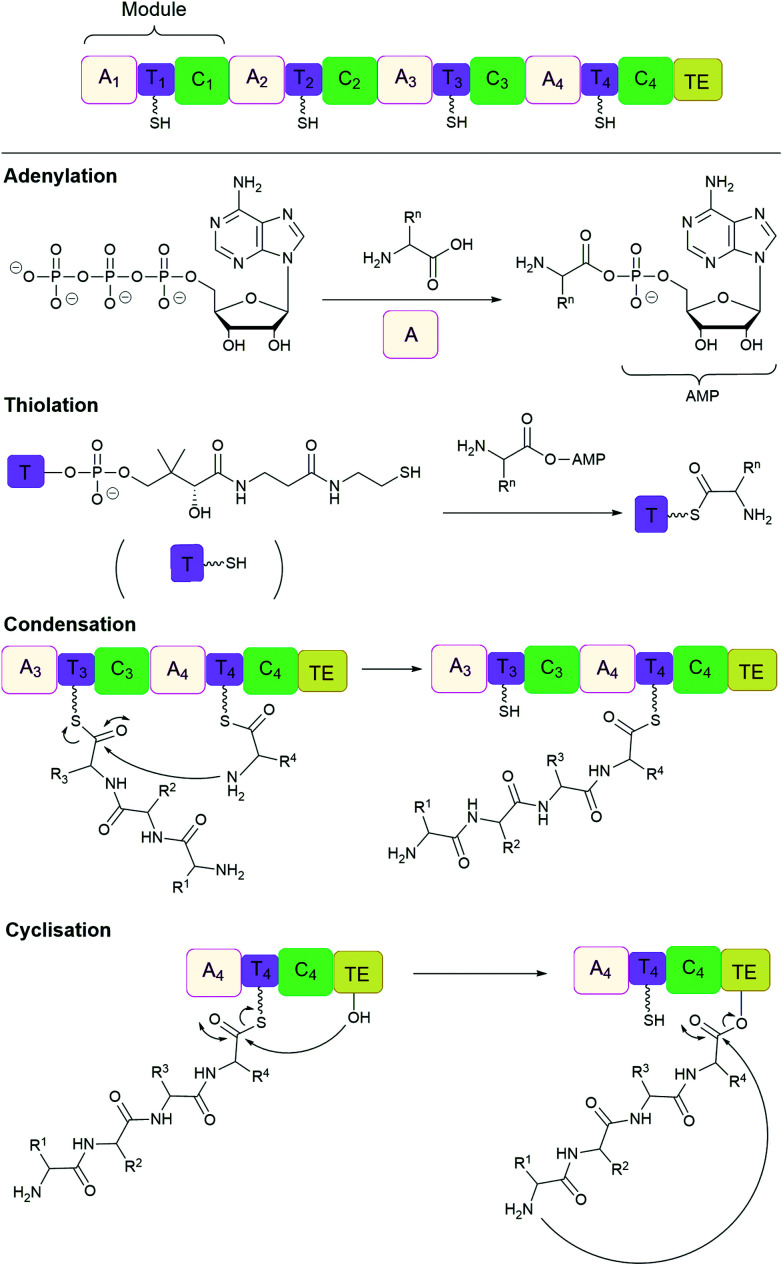
Non-ribosomal peptide synthesis by NRPS modules composed of adenylation (A), thiolation (T) and condensation (C) domains with a terminal thioesterase (TE) domain.

Many important natural cyclic peptides (and depsipeptides) are constructed by non-ribosomal peptide synthetases (NPRS). NRPS are large multifunctional enzymes that assemble one type of polypeptide without the need for cell ribosomal machinery and messenger RNAs. Each module of the NRPS is responsible for the incorporation of a specific amino acid building block. These modules are further divided into domains which catalyse a single reaction step.^131^ At minimum, a NRPS module is composed of three domains (Fig. 7):

(1) **Adenylation (A) domain** activates a specific amino acid by transesterification with ATP to generate the corresponding aminoacyl-adenylate.

(2) **Thiolation (T) domain** (also known as the peptidyl carrier protein) tethers the activated substrate to the enzyme through the formation of a thioester linkage.

(3) **Condensation (C) domain** catalyses the formation of a peptide bond between the activated acyl group and the free amino group of an amino acid on the neighbouring module.

In this way, the peptide chain grows in the N-to-C-terminal direction, until it is released by a **thioesterase (TE) domain** through either hydrolysis, oligomerisation or cyclisation, catalysed by the active site Ser-His-Asp catalytic triad.^132,133^ However, the large size (often 100 to 300 kDa) and complex multidomain structures of NRPS make their heterologous expression challenging, and has therefore lead to the development of alternative methods of production.^134^ For example, cell-free protein synthesis was employed for the *in vitro* reconstitution of the non-ribosomal cyclic peptide valinomycin gene cluster. As a result, valinomycin was produced in a yield of ∼30 mg L^−1^, comparable to that of native *Streptomyces* organisms.^135^

In addition, TE domains can also function as isolated enzymes. For example, cyclic tyrocidine A was generated from its linear precursor by the TE domain of tyrocidine synthetase.^136,137^ Synthetic peptide substrates are activated by the attachment of *N*-acetylcysteamine (SNAC) to the C-terminus. In this way, the natural tethering of the peptide chain, through the cofactor 4′-phosphopantetheine, is imitated. Furthermore, NRPS can be merged with SPPS for cyclising peptides immobilised on solid supports.^138^

The ability of NRPS to incorporate unnatural and d-amino acids, as well as carry out modifications such as epimerisation, methylation and reduction, results in large structural diversity of peptide products. Although reprogramming of these complex enzymes has so far yielded mixed results,^139^ the potential for the manipulation of NRPS remains great, especially with advances in the understanding of NRPS. For some dedicated recent reviews on this subject please refer to references.^140,141^

### 3.6. Remarks

Any enzyme that catalyses peptide bond formation can be potentially used for backbone cyclisation. Subtiligase, sortases, asparaginyl endopeptidases and their variants are some of the most popular choices due to their ability to generate the desired cyclic products in high yields. Enzymes, such as transglutaminase, that catalyse bond formation between amino acid side chain groups can also be employed. However, larger proteins may contain multiple enzyme recognition sites, resulting in increased off-target modifications or degradation of the protein substrate. This is particularly the case for enzymes that have relaxed substrate specificity (*e.g.* subtiligase derivatives, asparaginyl endopeptidases and transglutaminase). Moreover, the recombinant expression and purification of the enzyme, for example butelase 1 or NRPS, can be laborious.^104,142^ These considerations should be taken into account when using enzymatic approaches for cyclisation. Some of the key features of each enzymatic approach are summarised in Table 2.

**Table 1:** Chemical approaches for cyclisation

Method	Unnatural functionality^a^	Cyclisation mode	Applicable substrates	Applicable to recombinant materials	Use in living systems
Direct coupling	Often required in the starting material	Backbone	Peptides	X	X
Native chemical ligation	Incorporation of thioester motif	Backbone	Peptides, proteins	✓	X^b^
Ser/Thr ligation	Required	Backbone	Peptides	X	X
CyClick	Required	Backbone	Peptides	X	X
Traceless Staudinger ligation	Required in the starting materials	Backbone	Peptides	X	X
KAHA (type I and II)	Required	Backbone	Peptides	X	X
TAMM	Required	Side chain	Peptides, proteins	✓	✓
Cu-catalysed azide–alkyne cycloaddition (CuAAC)	Required	Side chain	Peptides, proteins	✓	X
Strain promoted azide–alkyne cycloaddition (SPAAC)	Required	Side chain	Peptides, proteins	X	✓
Cysteine disulphides	Not needed	Side chain	Peptides, proteins	✓	✓
Orthogonal disulphide pairing	Required	Side chain	Peptides	X	X
Disulphide stapling	Present in the products	Side chain	Peptides, proteins	✓	X

a Unnatural functionality refers to presence of any moiety that cannot be produced from 20 canonical amino acids.

b See section 4.1 for the recombinant extension to native chemical ligation (*i.e.* expressed protein ligation) which can be used in living systems.

**Table 2:** Enzymatic and protein tag approaches for cyclisation. All approaches are theoretically applicable to peptide and protein substrates prepared either chemically or recombinantly

Method	Cyclisation mode	Reaction motifs	Extra residues on the cyclic product	Use in living systems
Subtiligase variants	Backbone	Peptide ester/thioester + XX^a^	0	X
Sortases	Backbone	LPXTG + GG	6 (LPXTGG)	✓
Asparaginyl endopeptidase – butelase 1	Backbone	N/D-HV + G/L-X	3 (NXX)	✓
Asparaginyl endopeptidase – *Oa*AEP1	Backbone	NXX + X-F/I/L/M/W^b^	3 (NXX)	To be demonstrated
Microbial transglutaminase (*S. mobaraensis*)	Side chain	K + Q	0 or 2 (Lys and Gln)	✓
Protein tag – intein	Backbone	Split N- and C-inteins	0 or 1 (Cys)	✓
Protein tag – SpyTag/SpyCatcher	Side chain	Formation of an isopeptide bond between SpyTag/SpyCatcher	129	✓
Protein tag – SpyLigase	Side chain	SpyLigase catalyses isopeptide bond formation between SpyTag/KTag	23	To be demonstrated
Protein tag – SnoopTag/SnoopCatcher	Side chain	Formation of an isopeptide bond between SnoopTag/SnoopCatcher	124	To be demonstrated
Protein tag – SnoopLigase	Side chain	SnoopLigase catalyses isopeptide bond formation between SnoopTagJr/DogTag	35	To be demonstrated

a Subtiligase variants have very broad substrate scope but the identity of substrate residues in positions P1–P4 and P1′–P2′ influences the ligation efficiency. Generally, hydrophobic residues are preferred.

b *Oa*AEP1 can recognise a wide range of substrates, although its native substrates have the sequence NGL + GL.

## 4. Using a protein tag for cyclisation

Non-catalytic protein domains can also be used for cyclisation of peptides and proteins. These protein domains need to be fused to the polypeptide of interest, commonly achieved by genetic means. Consequently, these approaches are particularly suitable for production of cyclic peptides and proteins in living systems, which can be technically challenging by other methods.

### 4.1. Inteins

An intein is a protein domain that undergoes self-splicing.^143^ In this process, the intein excises itself from the protein and joins its flanking sequences, known as exteins, with a peptide bond. The splicing process is normally spontaneous, requiring only the correct folding of the intein to bring the extein termini into close proximity. It also does not require the presence of a cofactor or external energy source.

**Fig. 8:**
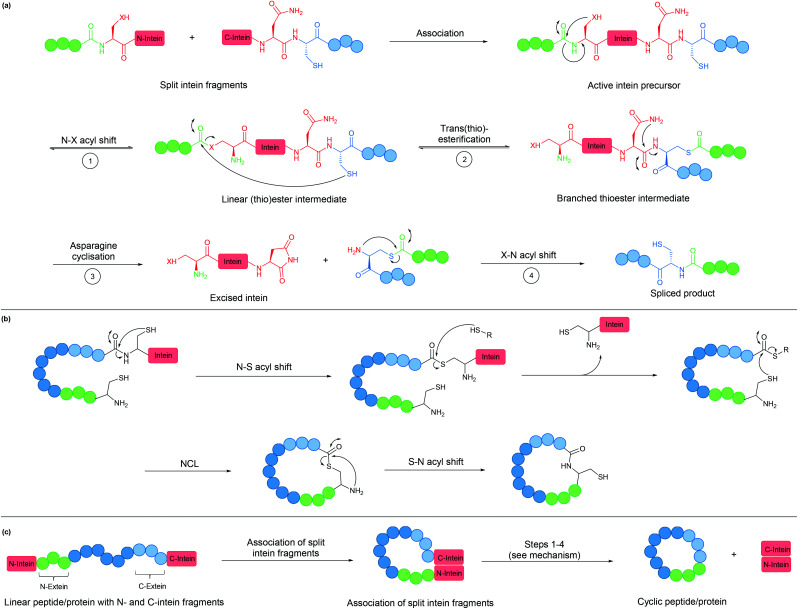
(a) Mechanism of intein splicing (X = O or S). (b) Cyclisation by expressed protein ligation. (c) Intein-mediated backbone cyclisation.

Mechanistically, the splicing begins with N–S or N–O acyl shift leading to the formation of a (thio)ester intermediate, followed by trans(thio)esterification between N- and C-exteins resulting in a branched intermediate. Next, intein excision proceeds through asparagine (or sometimes glutamine) cyclisation before S–N or O–N acyl shift take place to form the peptide bond between the exteins (Fig. 8a).

Inteins can be used to generate recombinant peptidyl fragments with a C-terminal thioester (Fig. 8b), which is required in native chemical ligation. This concept, known as expressed protein ligation (EPL), has greatly expanded the scope of native chemical ligation and has also been applied to cyclise large recombinantly produced proteins (*e.g.* β-lactamase).^22^

While the majority of inteins exist in a single contiguous form, such as those employed for EPL, some inteins naturally exist as two separate polypeptide chains. These split inteins undergo a *trans*-splicing mechanism, in which the N- and C-intein fragments first associate to form the active complex in the correct conformation before the splicing takes place (Fig. 8c).^143^ A prominent example is found in the *DnaE* gene of cyanobacterium *Nostoc punctiforme* PCC73102. This split intein is known as *Npu* DnaE. It exhibits fast splicing kinetics (*k* = 3.7 × 10^−2^ s^−1^)^144^ and good tolerance towards extein sequence variations,^145^ compared to another commonly used DnaE split intein from *Synechocystis* sp. PCC6803 (*Ssp DnaE*) which is more sensitive to variation in the extein sequence around the splice junction.^144,146^ By correctly positioning the two fragments of a split intein at the two ends of a peptide, a cyclic peptide is generated upon intein splicing. This strategy, commonly referred to as split-intein circular ligation of peptides and proteins (SICLOPPS), has been employed for backbone cyclisation of peptides and proteins in *E. coli*, yeast and mammalian cells.^23,147,148^

Overall, inteins are useful tools for backbone cyclisation that can be achieved in a (nearly) traceless manner, whereby only a single Cys/Ser residue remains at the ligation site after intein splicing takes place. With increased understanding and their wide spread occurrence in nature, new inteins have been engineered with improved properties, although limitations still remain.^149^ Most commonly, the introduction of an intein can lead to protein misfolding. The relatively large size of the intein can also lead to reduced yields from recombinant expression.^150–154^ Lastly, gene expression of the *Npu* DnaE split intein has been reported to be toxic to *E. coli*, an issue that was circumvented by the incorporation of a degradation tag to remove any spliced intein fragments or unspliced starting material.^155^

### 4.2. Protein tags for isopeptide bond formation

**Fig. 9:**
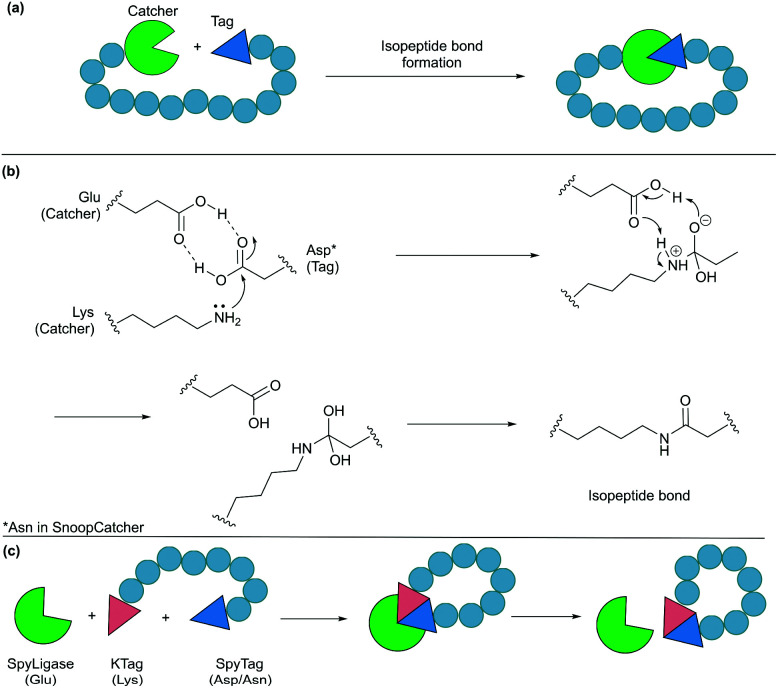
Cyclisation by a protein tag. (a) Isopeptide bond formation through a catcher and a tag. (b) Mechanism of isopeptide bond formation in SpyCatcher/SpyTag. (c) Catalytic version of SpyCatcher/SpyTag.

Formation of isopeptide bonds between amino acid side chains can be used to form circular polypeptides structures that are chemically stable and protease resistant. The CnaB2 domain of the fibronectin binding protein FbaB from *Streptococcus pyogenes* was found to contain an isopeptide bond between a Lys and an Asp residue. When splitting the CnaB2 domain into a 13-residue SpyTag peptide (containing the Asp) and a 116-residue SpyCatcher segment (containing the Lys), the two fragments were found to spontaneously and efficiently reconstitute *in vitro* and *in vivo* with an isopeptide bond formed between the Asp and Lys residues (Fig. 9a and b).^156^ Rapid isopeptide bond formation (*k* ∼10^3^ M^−1^ s^−1^) was observed under a variety of reaction conditions (4–37 °C, pH 5.0–8.0 with no requirement for specific anions or cations). The SpyTag/SpyCatcher partners have been used to cyclise different proteins including β-lactamase, dihydrofolate reductase, firefly luciferase and l-phenylalanine aldolase, all of which showed improved stability compared to their linear forms.^24,157,158^

Similarly, another isopeptide bond forming pair, known as SnoopTag (12 residues) and SnoopCatcher (112 residues), was developed by splitting the D4 Ig-like domain of adhesin RrgA from *Streptococcus pneumonia*.^159^ In this case, the isopeptide bond formation occurs between Asn and Lys residues and, importantly, it is orthogonal to the analogous SpyTag/SpyCatcher reaction. SnoopTag/SnoopCatcher was also employed to cyclise firefly luciferase.^24^ It was observed to confer improved stability to the cyclised product compared to the linear control, albeit enhancement was not as great as that observed with the SpyTag/SpyCatcher system.

The spontaneous formation of an isopeptide bond proves that SpyTag/SpyCatcher and SnoopTag/SnoopCatcher methods are practical and useful alternatives for protein cyclisation by genetically fusing the two components to the N- and C-termini of the target protein. Nevertheless, this approach leaves a large “scar” with >100 amino acids remaining in the cyclised products after ligation. This has been addressed by the development of a catalytic system composed of three parts, SpyTag, KTag and SpyLigase. To do this the 116-residue SpyCatcher was split into a 10-residue KTag and a 98-residue SpyLigase (the missing residues were omitted during the restructuring of the SpyCatcher fragment).^160^ In this system, shorter peptidyl fragments SpyTag and KTag, containing the reactive Asp and Lys residues, respectively, are incorporated into the substrate(s) of interest and subsequently crosslinked by the addition of SpyLigase (Fig. 9c). Similarly, SnoopTag/SnoopCatcher was redesigned into a 12-residue SnoopTagJr, a 23-residue DogTag and a 104-residue SnoopLigase.^161^ While the use of SpyLigase or SnoopLigase significantly reduces the number of amino acid residues left on the cyclic product, addition of >20 amino acids residues is still required by these means. Nevertheless, these approaches are all theoretically applicable in living systems as demonstrated with SpyTag/SpyCatcher in various examples.^162^

### 4.3. Remarks

In summary, protein tag cyclisation is a convenient method for cyclising recombinantly produced polypeptides, as the long protein tag sequences can be expressed alongside those of the peptide/protein of interest. In addition, cyclisation by protein tag methods generally proceed in a spontaneous and efficient manner. While intein-mediated cyclisation is considered traceless due to the excision of the intein sequence during splicing, isopeptide bond forming Tag/Catcher partners leave a large footprint at the ligation site, although it has been shown to aid protein stabilisation.

## 5. Considerations for cyclisation

Various methods for polypeptide cyclisation have been discussed along with their strengths and limitations which are summarised in Table 3. Unfortunately, no method is ideally suited for the cyclisation of all peptides and proteins. This section will focus on several key factors that should be considered when choosing a cyclisation approach.

**Table 3:** Summary of peptide/protein cyclisation techniques

	Cyclisation approach	Advantages	Factors to be considered
**Chemical**	Disulphide bond formation	• Cysteine residues easily introduced chemically or recombinantly (easily applied to smaller peptides and larger proteins)	• Not stable under reducing conditions (*i.e.* intracellular environment); could be addressed through the use of disulphide stapling reagents
		• Disulphide formation occurs readily under mild aqueous conditions	• Correct disulphide bond formation can be difficult to control and may lead to a mixture of products (addressed by orthogonal disulphide pairing but requires unnatural functionalities)
	Direct cyclisation	• Activation of the terminal carboxylic acid group allows reaction to proceed under mild conditions	• Nucleophilic and carboxylate amino acid side chains require protection to prevent side reactions
			• Limited to synthetic peptides
	CyClick	• Efficient, chemo- and stereoselective	• Requires an unnatural functionality in the starting material (C-terminal aldehyde); introduction can be achieved synthetically or through chemical modification (*e.g.* sodium periodate oxidation of Ser/Thr)
		• Can be performed at high concentrations without the formation of side products from intermolecular reactions	
	Native chemical ligation	• Reaction proceeds in aqueous conditions at neutral pH	• A C-terminal thioester is required which is introduced synthetically (addressed by expressed protein ligation)
		• Presence of chaotropic agents and reducing agents are tolerated and in some cases preferable	• Some NCL extensions involve the use of an unnatural thiol- or selenol-containing N-terminal amino acid in place of cysteine
		• Regio- and chemo-selective reaction	
		• A number of extensions have been developed for broader application (*e.g.* desulphurisation)	
	Staudinger ligation	• Traceless cyclisation	• Requires unnatural functionalities
		• Chemoselective towards the azide (protecting groups not required)	• Phosphinothiols only have limited solubility in aqueous solution
			• Glycine residues required at the ligation site
	α-Ketoacid-hydroxylamine ligation (Type II KAHA)	• Cyclises both longer and shorter polypeptides	• Requires unnatural functionalities in the starting material
		• O-Substituted hydroxylamine is water stable	• A homoserine residue is formed at the ligation site (although oxazetidine can be used in place of oxaproline to form a serine residue)
		• Chemoselective	• Reaction is relatively slow
		• Forms a native peptide bond	
	Cu-Catalysed azide–alkyne cycloaddition	• Efficient and regioselective	• Requires unnatural functionalities (azide and alkyne)
		• Requires only mild conditions and can be carried out in water	• Cu(i) catalyst must be generated *in situ* from Cu(ii) by the use of excess reducing agent and Cu-stabilising ligands
			• Cu is toxic to cells and so is not suitable for use *in vivo*
	Strain promoted azide–alkyne cycloaddition	• Circumvents the requirement for a copper catalyst	• Lacks regiospecificity (forms a mixture of 1,4-disubstituted products)
		• Rapid reaction under physiological conditions	• Cyclooctyne reagents are relatively costly
**Enzyme**	Subtiligase (and variants)	• Very broad substrate scope	• Promiscuity may result in off-target modificationsx
		• Effectively traceless	• Effectively traceless
	Sortase A	• Well-studied and understood enzyme	• Ligation is reversible as the LPXTG recognition sequence remains in the product
		• Mutants have been developed for improved activity	• Relatively low catalytic efficiency
		• Commercially available	• Ca^2+^ dependence, limited use *in vivo*
	Asparaginyl endopeptidase	• Exhibit high catalytic efficiency and requires a low enzyme-to-substrate ratio	• Short recognition sequence may lead to off-target modifications in larger proteins
		• Relatively broad substrate scope and short recognition sequence	• Recognition sequence remains in the product leading to reversibility of the reaction
		• Nearly traceless cyclisation (only Asx remains)	
	Microbial transglutaminase (*S. mobaraensis*)	• Calcium independent (in contrast to mammalian transglutaminases)	• Low substrate specificity
		• Resulting isopeptide bond is chemically and proteolytically stable	• Preference for glutamine residues is unclear
		• Tolerant to a broad range of reaction conditions	
		• Commercially available	
	Non-ribosomal peptide synthase	• Able to incorporate unnatural and d-amino acids, and can carry out modifications such as epimerisation, methylation and reduction	• So far reprogramming has yielded mixed results
		• TE domains can function as isolated enzymes	• Large size makes heterologous expression challenging
			• A deeper understanding of these complex systems required before their potential can be realised
**Protein Tag**	Intein (Expressed protein ligation and split intein mediated splicing)	• Overcomes size limitation of native chemical ligation	• Can result in low protein yields after recombinant expression
		• Almost traceless cyclisation (only Cys/Ser remains)	• Can lead to misfolding of the protein of interest
		• Does not require separate expression and purification	
	SpyTag/SpyCatcher (and SnoopTag/SnoopCatcher)	• Wide range of reaction conditions	• Leaves a large scar at the ligation site (addressed by the development of Spy- and SnoopLigase)
		• High yielding and fast	
		• Does not require separate expression and purification	

### 5.1. Distance between connecting residues

For protein substrates, it is important to consider the locations of the connecting residues. If located too far apart, the protein's complex folded structure may be disrupted as a result of strain, leading to loss of activity. It was found that in approximately 2000 representative proteins, 31% have their termini within 20 Å and 11% within 15 Å.^163^ Therefore, a large number of proteins could theoretically be amenable to head-to-tail cyclisation, as well as side chain-to-side chain cyclisation, assuming it is the side chain residues at, or close to the termini, that are to be linked together. Nevertheless, whichever mode of cyclisation is chosen, existing structural information can be used to select the most appropriate positions to take part in cyclisation. In cases where termini are located too distally for a suitable direct connection, linker sequences can be introduced to extend the termini, or bridging reagents of appropriate length can be used for connecting side chains.^164,165^ Interestingly, when cyclising granulocyte-colony stimulating factor using a split intein-mediated ligation approach, it was shown that the structure of the connector could be optimised to promote either enhanced stability or efficiency of cyclisation.^148^ When a longer connecter was employed, the increase in thermal stability was found to be greater (∼11 °C increase compared to a shorter linker). However, splicing efficiency was reduced, with unspliced starting material remaining. Although distortion caused by cyclisation is disadvantageous for improved stability and activity, it can be exploited. For example, cyclic luciferase was employed for real-time sensing of caspase-3 activity in living mammals.^166^ Cyclisation by split intein led to distortion of the luciferase structure and hence loss of bioluminescence activity. If N- and C-termini were linked by the caspase-3 recognition sequence (DEVD), the cyclised luciferase, in the presence of functional caspase-3, would be restored to its original active form and bioluminescence observed.

On the other hand, for peptide substrates without a specific three-dimensional conformation, factors such as ring size (*i.e.* length of linear precursor) and peptide sequence are known to affect cyclisation efficiency, as well as the reagents and conditions employed for cyclisation.^167^ Before cyclisation can take place, the reactive termini of the linear precursor must come into close proximity with one another. As such, cyclisation is favoured over intermolecular reaction. Various strategies have been developed to facilitate ring closure by pre-organisation of the linear peptide into a conformation predisposed to promote cyclisation.^28^ Generally, these involve the use of templates and modifications to the peptide sequence to increase flexibility or the introduction of turn-inducers (*e.g.* proline).^168^

### 5.2. Means to produce the polypeptide of interest

Another factor to consider is the method by which the polypeptide of interest is to be produced and purified. A chain of amino acids can be produced either chemically or recombinantly using cellular machinery. Solid-phase peptide synthesis is arguably the most common chemical means employed to generate peptides, whereby a solid support is used for the stepwise assembly of the peptide chain. After cleavage from the solid support, peptides are usually purified using reverse-phase HPLC. Larger polypeptides can also be synthesised chemically as smaller fragments which are later joined together for example using native chemical ligation.^34,35^ However, it is often preferable to prepare larger proteins by recombinant approaches using cellular machinery. This first requires molecular cloning for the introduction of the DNA molecule, containing the gene of the target protein, into cells. Once transformed, the recombinant DNA is transcribed into mRNA before translation into the target protein.^169^ Recombinantly produced polypeptides often require a number of chromatographic steps to achieve the desired level of purity. Most commonly these include affinity, ion-exchange and/or size exclusion chromatographies. It should be noted that affinity chromatography usually requires the incorporation of an affinity tag sequence (*e.g.* His-Tag used for immobilised metal affinity chromatography) into the polypeptide of interest, hence, an additional cleavage step to remove the tag may be required. Below, for each category of cyclisation approach, the relevance of chemical and recombinant preparation will be discussed.

Chemical cyclisation approaches often require non-native functionalities (see Table 1) or take place under non-physiological conditions (*e.g.* in organic solvent). As chemical synthesis is often favoured for the incorporation of non-native functionalities into specific positions within the polypeptide chain, it is perhaps the more convenient preparation method when using chemical cyclisation approaches. While it is also possible to introduce non-native functionalities by recombinant means such as genetic code expansion, there are more limitations in terms of amino acid substrates that can be introduced (see section 2.4). In addition, peptides can be synthesised in fully protected forms as required for some chemical ligation methods such as direct amide bond formation. On-resin cyclisation can also be carried out, which can be advantageous to solution phase approaches which often require high dilutions to minimise intermolecular reactions which generate dimer and oligomer side products. High dilution can also lead to long reaction times which in turn lead to epimerisation at the ligation site.^170^ Using the on-resin approach, the antibacterial peptides polymyxin B2, E2 and a derivative were cyclised. The peptides were anchored to the resin by the amine group of a lysine side chain.^171^ Upon removal of C- and N-terminal protecting groups, the peptides cyclised efficiently in good yields and exhibited antibacterial activities comparable to that of natural polymyxins. Alternatively, peptides can be anchored to the resin by the C-terminal carboxyl group through the use of safety-catch linkers.^172,173^ For example, the cyclisation of brachystemin A was carried out successfully using this approach.^174^

Preparation of enzymes is often essential when they are used as catalysts for cyclisation. However, this process can be time-consuming and labour-intensive, unless the enzyme is commercially available (*e.g.* sortase). Generally, enzymatic cyclisation can be applied to material produced by any means, although care should be taken with larger proteins which may contain multiple recognition sequences and thus result in off-target modifications causing degradation of the linear starting material and/or cyclised product.^121^

Protein tag cyclisation approaches such as intein and isopeptide bond forming Tag/Catcher partners are generally composed of sequences of ∼100 amino acid residues. It is therefore convenient to recombinantly express these long protein tag sequences alongside that of the polypeptide sequence of interest, as opposed to chemical synthesis followed by ligation to generate the required sequence. Moreover, it should be noted that while peptides are usually produced in a low yield when using recombinant production methods,^175^ the addition of the large flanking protein tag sequences required for cyclisation can facilitate their preparation by cellular machinery. When using intein-mediated cyclisation, these long sequences are excised during the cyclisation process and so do not remain in the product.^155^

### 5.3. Application-related factors

The type of cyclisation approach chosen can also depend on the eventual application of cyclised peptide or protein. Below, a few examples of application-related consideration will be discussed.

#### 5.3.1. Stability

Cyclisation is a useful technique for enhancing the stability of peptides and proteins and can therefore expand the scope of their application, for example as biocatalysts or as therapeutics. While there are numerous examples of proteins whose thermal stability has been increased as a result of cyclisation,^17^ the largest improvement towards heat treatment is often observed when cyclisation is carried out using isopeptide bond forming peptide/protein partners, in particular SpyTag/SpyCatcher.^157^ For example, SpyTag/SpyCatcher was demonstrated to stabilise PhyC phytase from heat induced aggregation at 100 °C and enabled the cyclised enzyme to be purified from cell lysate just by heating.^176^ Upon investigation using differential scanning calorimetry, it was found that isopeptide bond forming domains are likely conferring extra thermal resilience, on top of that achieved through cyclisation, by facilitating protein refolding after heat treatment.^176^

With regard to proteolytic stability, it may be useful to consider the mode of cyclisation, *i.e.* head-to-tail *versus* side chain cyclisation. Exopeptidases are enzymes that hydrolyse the terminal amide bonds of polypeptide chains. Thus, side chain cyclisation approaches which leave the termini free, for example disulphide bond formation, may leave the polypeptide susceptible to proteolytic degradation. For this reason, a head-to-tail cyclisation approach should result in improved resistance to proteolysis. For example β-lactamase, cyclised using an intein-mediated ligation approach, was shown to be resistance to treatment by carboxypeptidase Y, which hydrolyses C-terminal amide bonds.^22^ On a related note, proteolytic treatment can be used to test if cyclisation of a polypeptide has taken place, whereby digestion would only be observed if the linear form is present.^23^

#### 5.3.2. Library generation

Cyclic peptides are promising therapeutic candidates. In the process of identifying potential drug molecules through high-throughput screening techniques, a diverse library of cyclic peptide sequences needs to be generated. Some examples of popular methods employed for creating libraries of cyclic peptides include phage display, SICLOPPS and mRNA display, which will briefly be discussed below in the context of strengths and limitations of the cyclisation approach on the resulting cyclic peptide library.^177^

In phage display, bacteriophages that have been genetically modified to display unique peptides on the surface of their coat proteins, are screened for desired activity (*e.g.* selection by binding to a target molecule).^178^ By sequencing the phage DNA, the identity of the target-binding peptide can be determined. Phage display is a well-established and effective technique, and cyclic peptides can be generated by intramolecular disulphide bond formation between cysteine residues located either side of a randomised amino acid sequence (*i.e.* CX_*n*_C, where X_*n*_ is any number of any amino acids).^179–181^ However, cyclic peptides produced by this method are susceptible to reduction (*i.e.* linearisation) under reducing conditions. Alternatively, cyclic peptides can be generated using bioorthogonal reactions. For example, TAMM condensation was used to generate cyclic peptide library on bacteriophages, providing potent cyclic peptide binders to Bcl-2, Mdm2 and Keap1.^60^

Using SICLOPPS, cyclic peptide libraries can be prepared intracellularly.^23^ Here, a peptide library is created by randomisation of the extein sequence encoded by degenerate oligonucleotides.^182^ Upon excision of the split intein sequences, the termini of the extein sequence are ligated and the cyclic peptide generated *via* the formation of a native peptide bond. Cyclic peptide libraries have been generated using a variety of host cells, including *E. coli*, yeast and mammalian cells.^147,183,184^ This intracellular generation of cyclic peptides is advantageous, as it enables the use of cell-based screening against intracellular protein targets, as opposed to *in vitro* screening which does not always accurately reflect activity and function *in vivo*. Furthermore, it has been demonstrated that cyclic peptide libraries bearing non-canonical amino acids can be generated using this approach.^185^ However, like phage display peptide libraries, the maximum number of library members is limited by transformation efficiency of the host cells.^182^ In addition, the use of split inteins leads to certain extein sequence requirements and sometimes toxicity towards *E. coli* (see section 4.1).

A third strategy for cyclic peptide library generation is mRNA display. In this approach, the linear peptide is attached to its encoding mRNA sequence through a puromycin linker at the C-terminus.^186,187^ As such, highly efficient side chain-to-side chain or side chain-to-N-terminus cyclisation approaches are required.^188^ Disulphide bond formation between two cysteine residues is the most straightforward of these strategies. However, the use of bridging reagents and the incorporation of non-native amino acids^189,190^ can afford a wide range of possible cyclisation patterns, especially in the presence of multiple reactive residues.^191^ While non-specific cyclisation can make hit deconvolution difficult, increased library diversity and therefore investigation of a broader range of cyclic peptide scaffolds is advantageous.^192^ Recently, using an approach based on native chemical ligation, head-to-tail cyclisation of peptides compatible with mRNA display was reported, further broadening structural variety of mRNA display peptide libraries.^193^

#### 5.3.3. Size of footprint at ligation site

Broadly speaking, it is often preferable to have as little evidence of ligation remaining in the cyclised polypeptide product as possible (*i.e.* for cyclisation to be traceless). However, in most cases some form of footprint remains at the ligation site, whether it is a particular amino acid residue such as cysteine in NCL or Asx from the asparaginyl endopeptidase recognition sequence, the formation of a non-native group for example a 1,4-disubstituted triazole resulting from CuAAC, or a large protein tag sequence from SpyTag/SpyCatcher cyclisation. It should be noted, these scars are not always disadvantageous, although care should be taken that they will not interfere deleteriously with the intended function of the cyclised polypeptide.

## 6. Conclusion and perspectives

This review provides an overview of the approaches currently available for polypeptide cyclisation. Ideally, a cyclisation approach that is specific, traceless and applicable to both chemically and recombinantly prepared materials is desired. There is yet to be such an ideal method, and each reported method has its own strengths and limitations (Table 3). Generally, the choice of the most suitable approach largely depends on the sequence, how the material is produced and the desired application. While the production of chemically synthesised polypeptides is generally more laborious for longer sequences, they can be cyclised by a broader range of techniques. On the other hand, the preparation of polypeptides by recombinant approaches is technically simpler, but contains more restrictions (stereochemistry, incorporation of unnatural functionalities and polypeptide lengths). It is also noteworthy that many cyclisation approaches are theoretically orthogonal to each other and can be used to generate multiple cyclic structures simultaneously. Although this is a less explored direction, it may be an interesting area for future research with the potential to further expand the scope of application of cyclic polypeptides.

## Conflicts of interest

There are no conflicts to declare.
